# Multi-Channel Data Acquisition Card under New Acquisition and Transmission Architecture of High Frequency Ground Wave Radar

**DOI:** 10.3390/s21041128

**Published:** 2021-02-05

**Authors:** Yang Bai, Xin Zhang, Qiang Yang, Yong Yang, Weibo Deng, Di Yao

**Affiliations:** 1 Department of Electronics and Information Engineering, Harbin Institute of Technology, Harbin 150001, China; 18B905006@stu.hit.edu.cn (Y.B.); zhangxinhit@hit.edu.cn (X.Z.); yq@hit.edu.cn (Q.Y.); dengweibo@hit.edu.cn (W.D.); 2 The Ocean Observation Institute, Hainan Tropical Ocean University, Sanya 572022, China; 18B905030@stu.hit.edu.cn; 3 School of Information Science and Engineering, Harbin Institute of Technology at Weihai, Weihai 264209, China

**Keywords:** high-frequency ground-wave radar, acquisition card, DMA transmission, acquisition and transmission architecture

## Abstract

It is known that the data acquisition and processing system plays an important role in radar target detection system. In order to meet the requirements of real-time processing and accurate transmission of echo signals in high-frequency ground-wave radar (HFGWR) systems, a new acquisition and transmission framework utilizing the designed acquisition card based on the PCIe (peripheral component interconnect express) has been designed and is presented in this paper. The Xilinx FPGA (Field-Programmable Gate Array) chip Kintex7-XC7K325T is adopted as a hardware carrier in acquisition card. The hardware’s composition, analog front-end circuit, the DMA (Direct Memory Access) transmission, FPGA structure, ADC (Analog-to-Digital Converter) chip, and performance test of this card are showed and discussed. Currently, the acquisition card has been accomplished and applied in the practical system of HFGWR.

## 1. Introduction

With the development of electronic technology, data acquisition and processing system has been widely used in the fields of radar, optical fiber, telecommunications, sonar, and satellite remote sensing [1,2,3,4]. Reference [1] introduces a new data acquisition method, which realizes the acquisition of SCS (Symmetry Chirp Signal) signal in LEO Satellite Internet of things. In Ref. [2], a common radar data acquisition card is designed by using Xilinx IP core, and the driver design of the card is realized by WinDriver. In order to multi-channel image data that can be collected at the same time, a multi-channel data acquisition system based on Ethernet transmission is proposed [3]. A PXIe (PCI (Peripheral Component Interconnect) Express extensions for Instrumentation)-based multi-channel data acquisition system for a soft X-ray camera has been designed and is presented in [4]. Reference [5] designs the data acquisition system using ARM (Advanced RISC Machine) and Field-Programmable Gate Array (FPGA) architectures based on the application requirements of off-line multi-channel vibration acquisition and analysis system.

The HFGWR system, which works in the band from 3 MHz to 30 MHz, achieves over horizon and continuous monitoring of medium and large targets on the sea and reports the early warning intelligence by using the feature that radio waves of HF (high-frequency) wave band can conduct diffraction propagation on the sea. Due to its working wavelength and radio wave propagation characteristics, it has unique performance advantages, such as long detection distance, strong anti-stealth ability, cost-effectiveness, and so on. Meanwhile, the system can also establish tracks and tracking sequences for the target detected and report the location parameter, motion parameter, and other information of the target in real time. In addition, the HFGWR system can also extract information such as sea surface flow field, wind field, and wave spectrum, which can be used for marine environment detection, marine weather forecast, and sea state remote sensing. Therefore, it has a broader application prospects in monitoring the country’s exclusive economic zone and safeguarding national rights and interests.

Undoubtedly, during HFGWR detection, real-time processing and accurate acquisition and transmission of data will be crucial [6,7,8]. Under the new acquisition and transmission framework, in order to adequately collect and analyze the receiver’s output signals, a PCIe-based multi-channel data acquisition card that can convert analog signals into digital signals has been designed and is presented in this paper. Specifically, it relates to a new high-frequency ground wave radar signal acquisition and processing device, which is used to connect radar receiver and signal processor.

**Figure 1:**
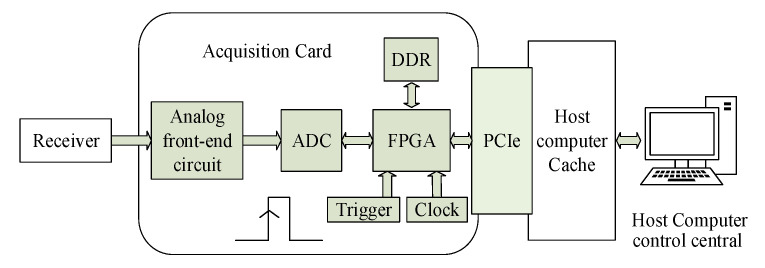
Block diagram of the new HFGWR acquisition system.

In this paper, we utilize the acquisition card to propose a new acquisition and transmission framework. A block diagram of the new proposed HFGWR acquisition system is presented in Figure 1. The system consists of receiver, analog front-end (AFE) circuit, Analog-to-Digital Converter (ADC), FPGA, trigger, clock, and DDR (Double Data Rage RAM). The receiver amplifies and filters the received analog signal and inputs the signal to the acquisition card through SMA (Subminiature version A) transmission line. The acquisition card has two acquisition modes; one is to directly acquire the IF (intermediate frequency 75 MHz) signal from the receiver output, that is, direct acquisition mode. The other is to collect the IF signal after DDC (digital down conversion), namely DDC mode. At last, DMA transmission mechanism transfers the buffer data to the upper computer of the signal processor for subsequent operations.

The acquisition and transmission framework proposed in this article obviously requires the design of an independent acquisition card. In order to ensure that the performance of the entire radar system is the same as the original framework, it is necessary to make specific requirements for the performance of the designed acquisition card. The following will introduce the design advantages in detail and test the performance of the acquisition card.

## 2. Processing Flow and System Advantages

### 2.1. Signal Processing Flow

The signal processing flow of the acquisition device in this article is as follows. The output signals of the receiver, which are HF narrow-band signals, are first amplified and balanced by the analog front-end circuit. Then, the ADC convert the analog signals into digital signals. Secondly, using FPGA to down convert the digital signals output from the ADC and caching the processed data into DDR. When the FPGA receives the trigger signal, the processed signals are transmitted to the host computer cache by PCIe interface through DMA [9,10,11,12]. Finally, the host computer control-central, which is adopted to perform signal processing by using uploaded data, obtains the distance, velocity, and orientation of the target to locate and track.

### 2.2. Advantages of Proposed Framework

In the previous HFGWR system, data acquisition and transmission are completed by the radar receiver and the optical fiber card respectively. When the antenna receives the echo signals, firstly, the receiver amplifies and filters the received antenna echo signals. Secondly, the ADC convert the analog signals into digital signals and then FPGA down convert the digital signals. Eventually, the processed data are sent to the signal processing machine through the optical fiber. Since the fiber card is also integrated into the receiver, all the above processes are completed inside the receiver. Therefore, this frame structure has some disadvantages, the receiver system is obviously very large and complex and the production and debugging of the optical fiber card inside the receiver is quite difficult and complicated [8]. In addition, the original data of the echo signals cannot be obtained in the host computer, in the debugging process of the actual system, it is difficult to determine which part caused the problem.

Compared with the traditional acquisition system, the proposed acquisition and transmission structure by utilizing acquisition card perfectly solves the shortcomings of the previous system. Due to the unique frame, the function of amplification and filtering and the analog-to-digital conversion of the receiver are separated, which reduces the complexity of the receiver system. And SMA transmission line is used to replace the role of the optical fiber card, which simplifies the process of data packaging, unpacking, and communication transmission. Meanwhile, using the direct acquisition mode of the acquisition card, the original data of the echo signal can be obtained in signal processor, so as to facilitate the inspection and investigation in the system [13,14,15,16,17,18,19]. Moreover, the control and parameter configuration of the acquisition card can be implemented manually in the signal processor, which makes it flexible and convenient to debug the system.

## 3. Hardware Structure Composition

**Figure 2:**
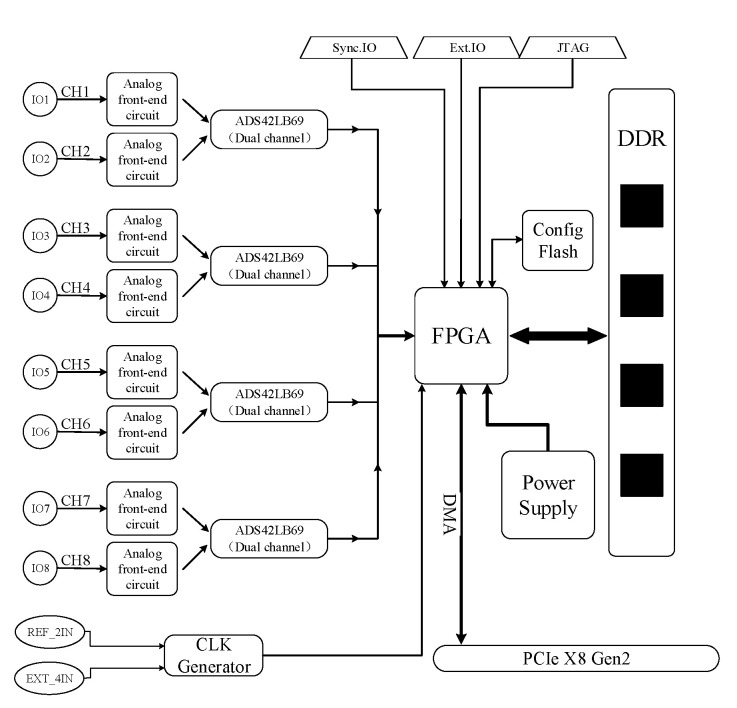
The structure of multi-channel data acquisition card.

In this part, the components of this acquisition card will be described in detail [9]. The structure of the multi-channel data acquisition card based on PCIe is shown in the Figure 2. It has a complex frame architecture with many input and export ports. The designed data acquisition card has eight input signal channel ports, four external trigger input ports, one external reference clock input port, and one external sampling clock input port. In addition, four dual-channel ADC chips are adopted to digitize the output signals of the 8-channel analog front-end. Moreover, it also has a JTAG (Joint Test Action Group) interface for testing FPGA internal chip and an 8-wire PCIe interface. Compared with multiplexing scheme, the designed card structure can avoid the problem of signal crosstalk [20,21,22,23,24]. The following is a brief introduction to the characteristic of each part.

### 3.1. Analog Front-End Circuit

**Figure 3:**
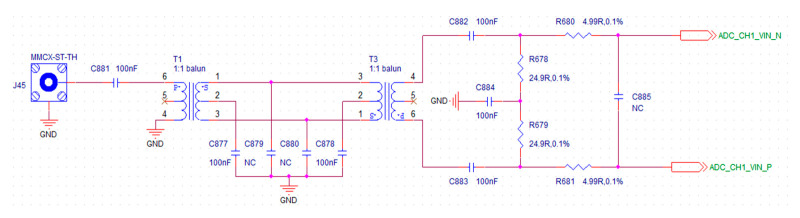
The analog front-end circuit diagram.

Considering that the high frequency analog signal is susceptible to signal interference in the muti-channel data acquisition card, in order to avoid this interference, the differential signal transmission is adopted in the system. It requires single ended signal changes to differential signal before the analog signal enters the ADC, so a differential amplification circuit is added before the ADC circuit. This is the role of the analog front-end circuit and one of the technical innovations of this acquisition card. The designed analog front-end circuit diagram is shown in Figure 3.

When the differential signal encounters interference, the two lines of the differential will be affected at the same time, but the voltage difference does not change a lot, so it has better anti-interference performance than the single ended signal. The differential amplifier circuit of analog front-end, which not only amplifies and equalizes the input RF signals, but also eliminates the high-order harmonics and improves power factor. Therefore, it has almost no interference among the multiple signals acquisition channels, nor does it have interference between the data acquisition card and the host computer during data transmission.

In addition, the AC coupling mode is adopted in data acquisition card, which removes the DC component and retains the AC component by using the DC isolation capacitor. Therefore, frequency of the input analog signal must be higher than 100kHz or it will be filtered out. The low frequency component is effectively prevented from entering the HF ground wave radar system.

### 3.2. ADC Circuit

The ADC chip connects the output of analog front end and the input of FPGA, which plays a critical role in whole acquisition card. Its input voltage range has to meet the output voltage range of the analog front-end circuit. Simultaneously, the sampling rate of the ADC must meet the Nyquist sampling rate, and the output of ADC should make the FPGA read. The commonly designed ADS42LB69 (16 bit), which is a family of high-linearity, dual-channel, 250-MSPS, analog-to-digital converters supporting DDR LVDS output interfaces, are used in this acquisition card [21].

**Figure 4:**
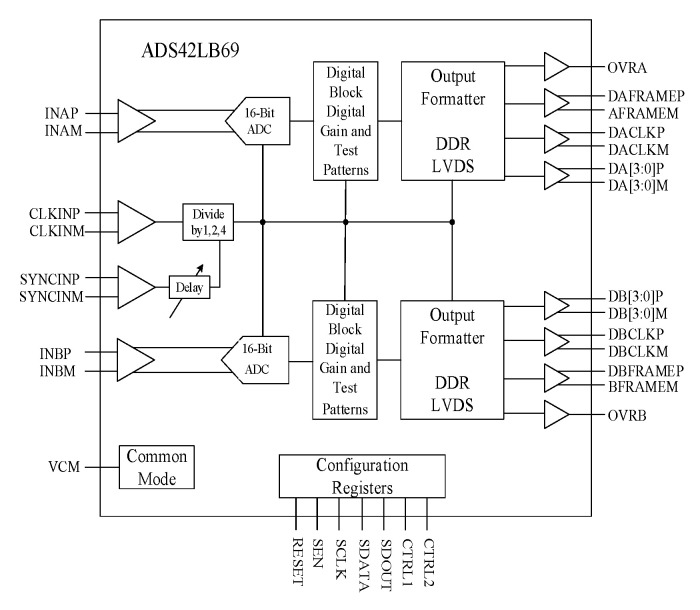
The simplified schematic of ADS42LB69.

The ADS42LB69 provides excellent spurious-free dynamic range (SFDR) over a large input frequency range with low-power consumption. Therefore, on the basis of ensuring the performance of the system, four dual channel ADS42LB69 chips are selected to realize 8-channel signal acquisition of radar system. A simplified schematic of the ADS42LB69 is presented in Figure 4 and Table 1 shows its pin functions.

### 3.3. FPGA

**Figure 5:**
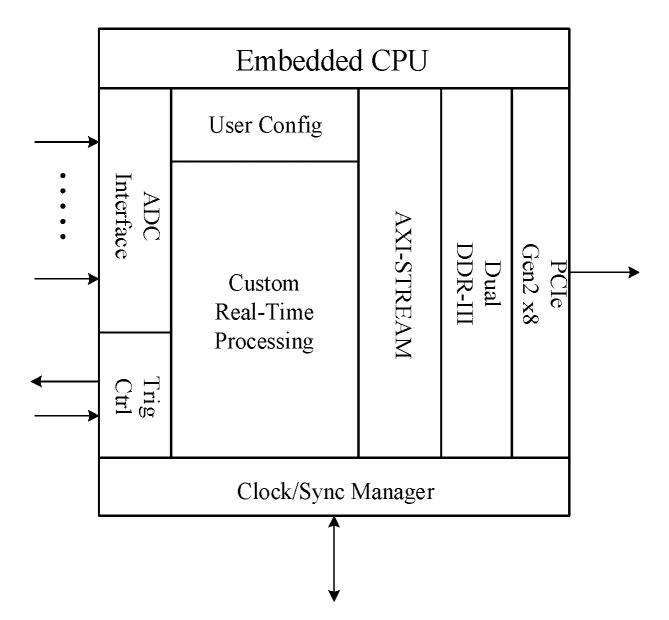
The internal structure diagram of Field-Programmable Gate Array (FPGA).

In the field of high-speed digital design, FPGA is widely used in high-speed data acquisition card because of its high clock frequency, small internal delay, many interfaces, and flexible use compared with traditional MCU (micro controller unit). In this acquisition card, a Xilinx FPGA chip Kintex7-XC7K325T, which has stable operation and strong anti-interference ability, is adopted to collect the receivers output signals (the amplitude ranges from −1.5 V to 1.5 V and the frequency ranges from 100 KHz to 100 MHz). It has some functions such as data merging, caching, and information statistics. It completely meets the requirements of high frequency ground wave radar system for data acquisition. As shown in Figure 5, the FPGA internal structure mainly include seven aspects: trigger control, ADC interface, clock manager, embedded CPU (central processing unit), AXI-Stream, DDR, and PCIe interface [22].

**Figure 6:**
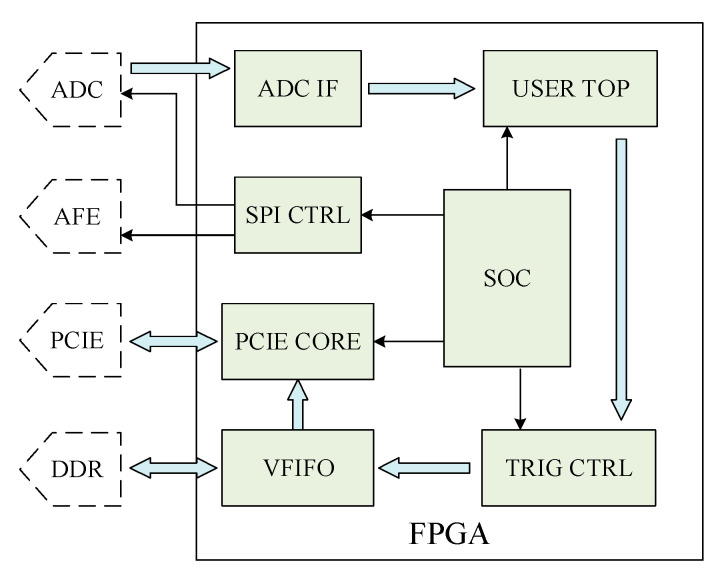
The internal logic interface diagram of FPGA.

The FPGA internal logic diagram is shown in Figure 6, including AD module, user module, trigger control module, PCIe module, SPI module, VFIFO module, and SOC module. AD module connects with AD chip and receives data collected by ADC through LVDS bus.

The user module has the function of logic redevelopment and provides the basic code of register reading and writing. The trigger control module is used to realize various trigger modes in the system and package and transmit collected data to the DDR module through the AXI-stream bus. The PCI-E module is mainly used to realize DMA data transmission between PC and FPGA. The SPI module is a SPI main controller, which can control all kinds of chips of the analog front-end and relays. The DDR module calls the kernel of Xilinx to drive two sets of DDR3 on the board and combines these two sets of DDR3 interface to virtual two sets of FIFO interface. The SOC module mainly includes soft core processor, AXI bus controller, and register interface of trigger control module and SPI module.

**Figure 7:**
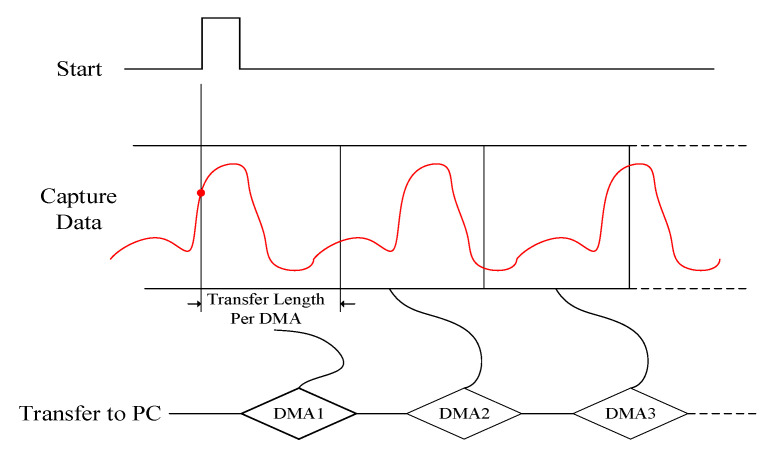
The schematic diagram of data acquisition and transmission.

A lot of logic programming are implemented by Verilog HDL in FPGA—for example, the serial data to parallel data conversion, the PCIe transmission bus protocol, the data cache implementation, the logic function debugging, the performance testing, and so on. Figure 7 shows a schematic diagram of data acquisition and transmission using FPGA. When the FPGA receives the trigger signal, the acquisition and transmission system will continuously transmit data to the memory of the host computer while continuously capturing data. The processed data are transmitted to the Host computer through the PCIe connector using DMA transmission mechanism. This architecture can ensure the real-time of data acquisition and transmission, so as to ensure the real-time processing of the system.

### 3.4. Connection between FPGA and PCIe

**Figure 8:**
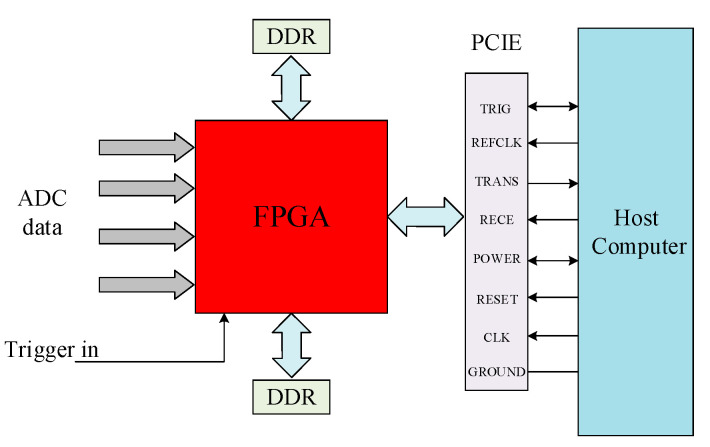
Connection circuit diagram of FPGA and PCIe.

The PCIe connector is used to transmit the FPGA’s output data to the host computer with transmission speeds that can reach up to 4Gb/s. The connection circuit diagram of FPGA and PCIe is presented in Figure 8. The pins’ functions of the PCIe connector are presented in Table 2.

### 3.5. DMA Transmission Architecture

In this system, PCIe bus and DMA mechanism are used to transfer data to the host computer. This is because there are still some problems in using the PCIe IP core alone to transmit data. Firstly, the CPU resources of the host computer will be occupied all the time in the process of data transmission, making the host computer itself unable to perform other operations. Secondly, when using PCIe to transmit a large amount of data, it will transmit many times, which wastes a lot of time in initiating and ending transmission, resulting in the decline of average transmission rate. The DMA mechanism solves these problems very well and provides guarantee for the HFGWR system to report the position and motion parameters of the target in real time [25,26,27,28]. The DMA controller uploads the fixed-size data to the memory space of the host computer designated by the CPU. The CPU is not required in the transmission process, so the computing resources of the host computer are saved. After the data collected and processed is buffered in the on-board memory, FPGA first moves data to the PC memory through the internal DMA engine, then the CPU carries the block data to hardware cache by raid controller, and finally stores it in the disk array. This is another technical innovation of this acquisition card.

**Figure 9:**
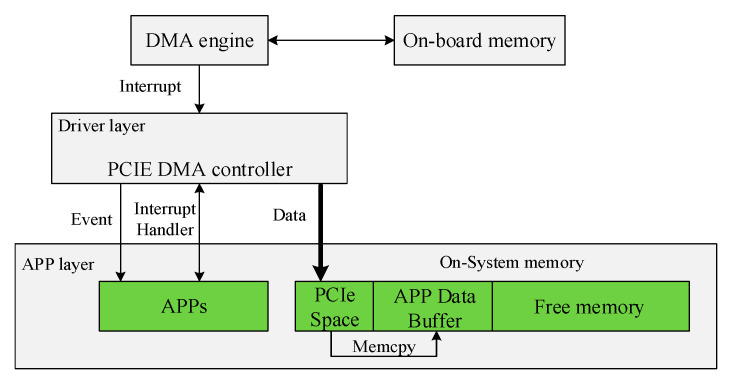
The schematic diagram of DMA transmission architecture.

In order to achieve the highest transmission efficiency, data transferring is carried out automatically by means of interruption. Figure 9 is the schematic diagram of DMA data transmission architecture. In the following, the DMA transfer process will be clearly presented.

Firstly, the DMA engine sends out an interrupt signal in the process of data transmission. Secondly, the driver layer responds to the interrupt and sends an event signal to the app layer. Then, after receiving the signal, APPs transfers a certain amount of data from PCIe space to app data buffer for further processing and then returns a signal to driver layer to inform that the next DMA operation can be started. Finally, it can continuously read data from on-board memory to PC memory.

### 3.6. Clock and Power Supply

**Figure 10:**
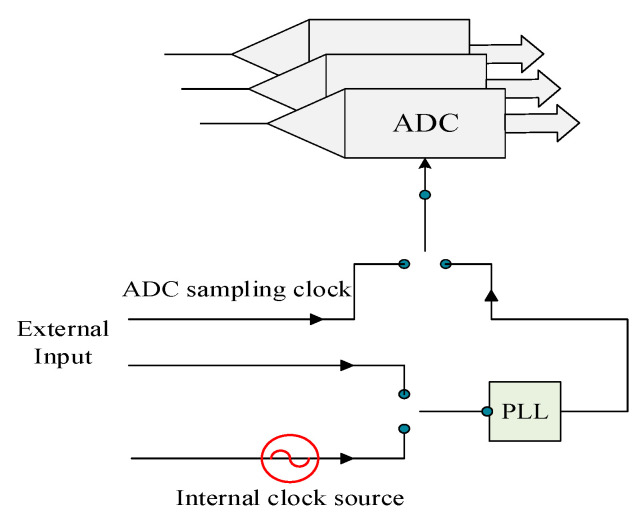
Diagram of clock source.

As is shown in Figure 10, there are two sources of reference clock: one is from the internal default clock source (100 MHz), the other is from external input. PLL (phase locked loop) multiplies the internal sampling clock to the frequency required by ADC. This design ensures that the working frequency of the card is the same as the local oscillator frequency of the receiver. Moreover, external input signal frequency also can be directly used as ADC sampling clock.

The normal and stable operation of a system is inseparable from a stable and reliable power conversion module. The current power supply schemes of the acquisition card have been relatively mature, which are not described in detail in this paper. A stabilized voltage supply is used to provide 12 V, and the power conversion module are adopted to convert the 12 V to the required supply voltages, such as ±5 V, 1.2 V, 2.5 V, and 3.3 V.

## 4. Performance Test of Acquisition Card

**Figure 11:**
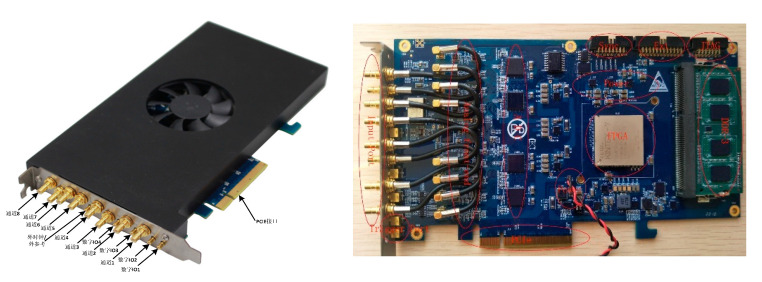
The designed data acquisition board.

**Figure 12:**
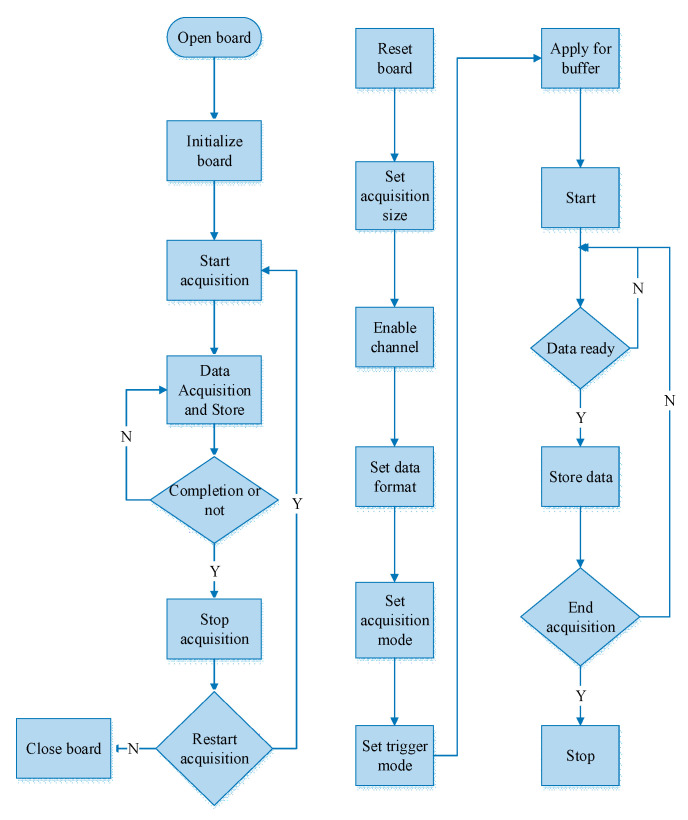
The Flow chart of acquisition card.

The Photograph of the designed data acquisition card and its workflow are shown in Figure 11 and Figure 12 respectively. The test parameters are given in Table 3 and the test results are shown below. All parameters of acquisition card can be configured and changed artificially in the host computer, which is also the key point of this system superior to the previous system.

Firstly, we use the analog signal generator to generate a single-frequency sinusoidal signal with a frequency of 75.01 MHz and an amplitude of 500 mV. Then the generated signal is divided into 8 parts equally by the power divider and input into 8 channels of acquisition card. Lastly, through the signal analysis of 8 channels, we obtain the performance parameters that our system cares about.

**Figure 13:**
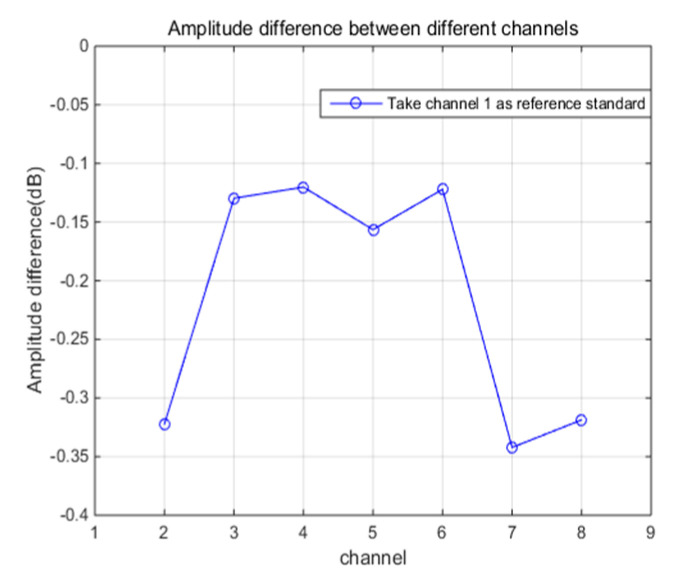
Amplitude errors between channels.

**Figure 14:**
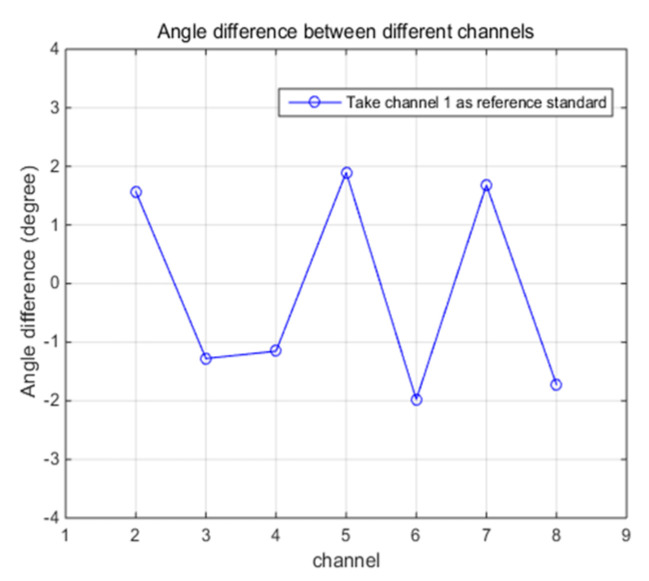
Angle errors between channels.

The test results of amplitude and angle error between channels are shown in Figure 13 and Figure 14. From the figures, we can find that the amplitude error between channels is within 0.35dB and the angle error is not more than 2 degrees. Digital beamforming technology (DBF) is used to estimate the azimuth of target in HFGWR; however, the main lobe width is limited by the array aperture and wavelength, the angle resolution of the radar is about 5 degrees [29,30]. Therefore, angle and amplitude errors are within the acceptable range.

**Figure 15:**
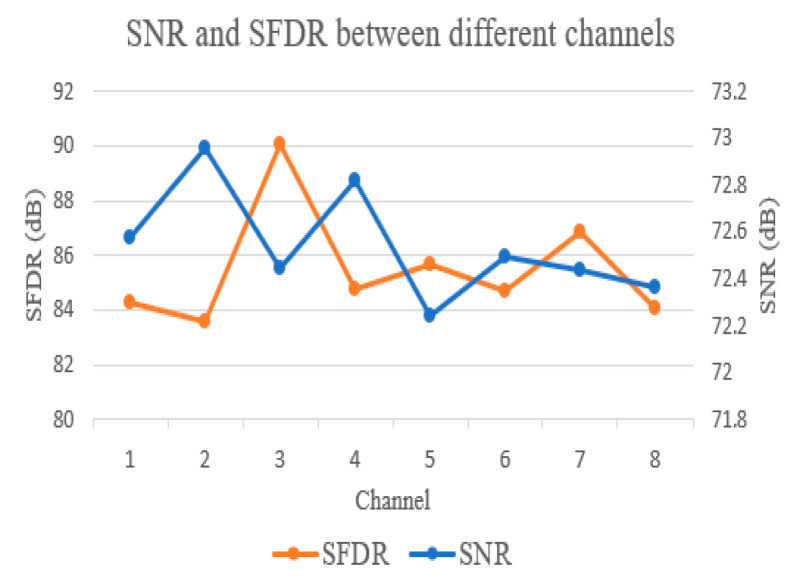
SNR and SFDR of acquisition system.

**Figure 16:**
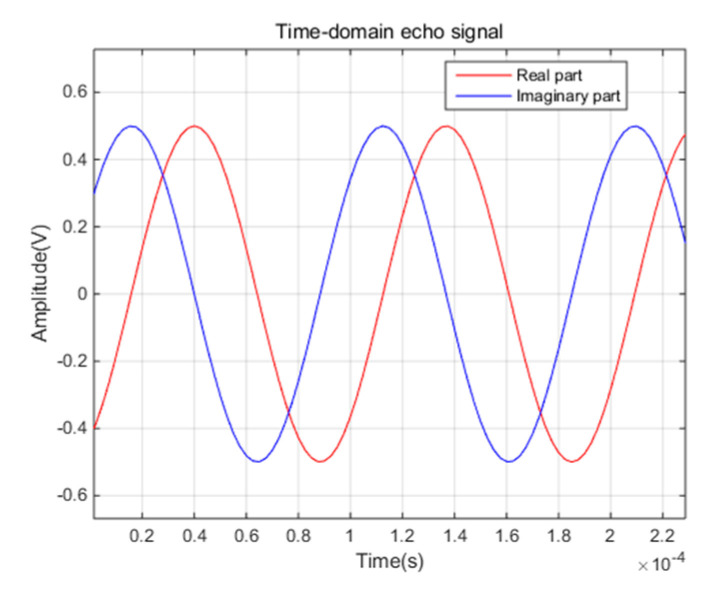
Waveform of echo signal.

The test results of SNR (Signal to Noise Ratio) and SFDR (Spurious-free-dynamic-range) between channels are shown in Figure 15. It can be seen that the SNR and SFDR of the acquisition card is about 72dB and 87dB. In HFGWR, the interference within the bandwidth will have a great impact on the detection of small targets, which may lead to the failure to detect the targets. According to the experience from the actual system, the maximum interference is less than 70 dB, which shows that the performance of SNR and SFDR is high enough. In addition, the waveform of echo signal after digital down conversion is presented in Figure 16. Hence, one can see that the performance of this acquisition card can fully meet the requirements of HFGWR system.

## 5. Discussion

As depicted by test results given in Section 4, the designed acquisition card can meet the requirements of real-time processing and accurate transmission of echo signals in a new acquisition and transmission framework of high-frequency ground-wave radar (HFGWR). The amplitude and phase error between acquisition card channels are within the acceptable range of the system and the errors between channels can be compensated by system simulation without affecting the accurate estimation of the target. Compared with the previous acquisition and transmission architecture, the proposed framework can not only reduce the complexity of the receiving system, but also obtain the initial data of the echo signal and facilitate the debugging of the system.

Furthermore, it is not only applicable for HFSWR system, the proposed data acquisition framework can also be used in other multi-channel radar systems. However, the digital to analog conversion accuracy of the acquisition card needs to be further improved and the parameters of the filter cannot be adjusted as needed.

## 6. Conclusions

In this paper, we utilize a multi-channel data acquisition card to propose a new acquisition and transmission framework, achieving the real-time acquisition of data and solving the shortcomings of the previous system. Importantly, a differential transmission mode is adopted to reduce the signal crosstalk and DMA architecture is used to reduce the waste of resources in the design process of acquisition card. Meanwhile, through the performance test of the acquisition card, it is showed that the echo signal has been acquired and processed by proposed system with accuracy. At present, the acquisition card has been accomplished and applied in the proposed practical system of HFGWR.

## Figures and Tables

**Table 1:** Parts of Pin functions of the ADS42LB69.

Name	Description	I/O
INAP/INAM	Differential analog input for channel A	I
INBP/INBM	Differential analog input for channel B	I
VCM	Common-mode voltage for analog inputs	O
CLKINP/CLKINM	Differential clock input for ADC	I
SYNCINP/SYNCINM	External sync input	I
DAP/DAM	4-bit DDR LVDS output interface for channel A	O
DBP/DBM	4-bit DDR LVDS output interface for channel B	O
DACLKP/DACLKM	Differential output clock for channel A	O
DBCLKP/DBCLKM	Differential output clock for channel B	O
OVRA/OVRB	Overrange indication channel A/B	O

**Table 2:** Pin functions of the PCIe connector.

Name	Description
TRIG	PCIe TRIG is a bidirectional trigger signaland it is used to trigger the PCIe connector
INBP/INBMPOWER	Supplies power to the PCIe card
TRANS	Signal transmission pin of the PCIe connector
RECE	Signal reception pin of the PCIe connector
RESET	Reset signal
CLK	Clock Request Signal
REFCLK	100 MHz Reference clock
GROUND	Zero voltage reference

**Table 3:** Test parameters of acquisition.

Parameters	Value
Input Signal Frequency (MHz)	75.01
Input Signal Amplitude (mV)	500
Sampling rate (MHz)	250
Acquisition mode	DDC
Bandwidth (KHz)	50
Output sample rate (KHz)	400
Trigger	External trigger
Clock	internal default clock source
